# Functional Test Scales for Evaluating Cell-Based Therapies in Animal Models of Spinal Cord Injury

**DOI:** 10.1155/2017/5160261

**Published:** 2017-10-04

**Authors:** Woon Ryoung Kim, Minjin Kang, Heejoo Park, Hyun-Joo Ham, Hyunji Lee, Dongho Geum

**Affiliations:** Department of Biomedical Sciences, Korea University College of Medicine, Seoul 136-705, Republic of Korea

## Abstract

Recently, spinal cord researchers have focused on multifaceted approaches for the treatment of spinal cord injury (SCI). However, as there is no cure for the deficits produced by SCI, various therapeutic strategies have been examined using animal models. Due to the lack of standardized functional assessment tools for use in such models, it is important to choose a suitable animal model and precise behavioral test when evaluating the efficacy of potential SCI treatments. In the present review, we discuss recent evidence regarding functional recovery in various animal models of SCI, summarize the representative models currently used, evaluate recent cell-based therapeutic approaches, and aim to identify the most precise and appropriate scales for functional assessment in such research.

## 1. Introduction

Spinal cord injury (SCI) is defined as damage to any spinal cord segment or nerve root. Such injuries often lead to permanent functional changes, such as paralysis or diminished muscle strength, movement, or sensation below the injured region. Thus, the pathological symptoms of SCI can vary depending on the site of injury and severity of the damage. As the annual incidence of SCI due to vehicular accidents or falls continues to increase worldwide [1, 2], the need to develop novel therapeutic strategies for SCI remains urgent in the field of spinal cord research. Due to the epidemiology and severity of SCI, various therapeutic techniques and biomaterials have been proposed [3, 4]. Most of these potential therapeutic agents are primarily tested in various animal models of SCI (e.g., rats, cats, dogs, and nonhuman primates) [5, 6]. In addition to the inherent advantages of animal models for evaluating the therapeutic effects of potential treatment strategies, such models are highly useful in assessing the degree of sensory/motor impairment following injury.

Among the four regions of the spinal cord (i.e., cervical, thoracic, lumbar, and sacral), the cervical and thoracic regions are most frequently studied in animal models of SCI. Injuries to the cervical spinal cord affect most of the body, including the arms and legs [1, 7], while those to the thoracic spinal cord and associated nerves result in trunk instability and abnormal movement of the lower extremities. In both rodents and primates, the anatomical locations of these regions allow for easy induction of SCI, analyses, and therapeutic manipulation involving cellular or biomaterial-based grafts. Based on the specific deficits observed, various behavioral tests have been developed to assess motor function and recovery following treatment in animal models of SCI [8–10].

The present review discusses recent evidence regarding functional testing in various animal models of SCI, the most suitable animal models for evaluating SCI, and current therapeutic challenges in SCI treatment. As several studies have highlighted the need for a well-developed, objective, and universal rating scale of functional impairments in rat models of SCI—such as the Basso, Beattie, and Bresnahan (BBB) scale [8–11]—we focused on suitable scales for assessing behavior, functional deficits, and therapeutic effects following a certain period of recovery or treatment.

## 2. Animal Models of Spinal Cord Injury

### 2.1. Traumatic Lesions

**Figure 1:**
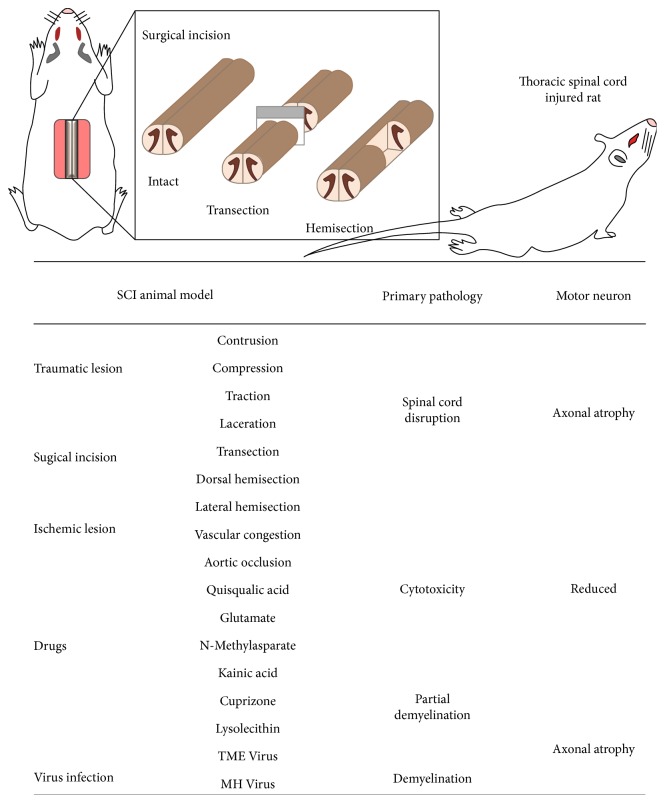
Development of animal models of spinal cord injury. (Upper illustration) methods for inducing spinal cord injury via surgical incision include transection and hemisection, both of which can be accomplished using a surgical knife (left). Hind limb paralysis following thoracic spinal cord injury in rats (right). (Lower table) animal models categorized by lesion method (left), the resulting primary pathology (middle), and final result of affected motor neurons (right).

Since SCI frequently occurs following physical trauma (e.g., vehicular accident and falls), animal models of SCI are frequently developed using external stimuli such as dropped weight or clip compression (summarized in Figure 1). In animal models, traumatic SCIs are typically induced via contusion, compression, traction, or laceration. Among these, spinal contusion is most frequently utilized, as the size and severity of injury can be accurately adjusted and replicated using a well-defined weight-drop impactor [12]. The contusion method, which was first used to develop animal models of thoracic SCI, can cause severe damage and paralysis, which can be life-threatening when applied to the cervical region. Thus, hemicontusion is preferred for models of cervical SCI [13]. In clip compression models, the size and severity of damage can consistently be controlled by adjusting the time, forces, and angle of compression. Since compression can result in severe injury without transection, this method is advantageous for investigating variations in recovery over time. Tractive SCI can be induced using a specialized spinal retractor, which allows the experimenter to control the length and duration of traction [14]. Such methods not only damage the structure of the spinal cord but also produce severe reductions in neuronal numbers. SCI via laceration results in severe irregular axonal damage, which mimics spinal injury due to a physical accident. Laceration is induced in animal models of SCI using the oscillating blade of a novel device known as the Vibraknife, which allows for precise control of the depth and size of the lesion without laminectomy [15].

### 2.2. Surgical Incision

Surgical incision is often utilized to develop models of SCI following penetrating injuries, such as knife wounds. Since the ends of the spine can be incised clearly and accurately using a blade, such models have been widely utilized in the investigation of neuronal regeneration and tissue engineering procedures (Figure 1, upper illustration). Since traumatic injuries do not completely sever the spinal cord, the transection method is more suitable for mimicking the symptoms of “complete” SCI, in which patients exhibit total and permanent loss of function below the injured site. Thus, the transection SCI model has been utilized to explore the function of each spinal segment and to evaluate functional and anatomical regeneration following complete transection [7, 16, 17]. In addition, partial transection via unilateral, dorsal, or ventral hemisection is highly useful in the investigation of therapeutic challenges such as transplantation. Both functional and anatomical alterations can be examined more precisely by comparing the ipsilateral and contralateral regions [18–20].

### 2.3. Ischemic Lesions

The spinal cord and spinal canal can be severely injured due to ischemia, which can be induced by either vascular congestion or aortic occlusion. Recent animal studies have frequently utilized photochemical ischemia to induce vascular congestion [21–23]. In this approach, following systemic injection of a dye such as Rose Bengal, spinal cord vessels are focally irradiated with an argon ion laser (560 nm). The ensuing photochemical reaction leads to vascular stasis without laminectomy, thereby resulting in tissue infarction and functional deficits. In contrast, aortic occlusion can easily be induced using an occlusion catheter [24], which allows for control of both the duration and severity of occlusion.

### 2.4. Drug-Induced Lesions

The primary advantage of a drug-induced model of SCI is that both tissue damage and functional deficits can be induced via local injection, without the need for laminectomy. Since both lesion area and functional outcomes are highly reproducible, the drug-induced SCI model has been widely utilized in this field of research. Local injection of excitotoxic drugs such as quisqualic acid, glutamate, N-methyl-D-aspartate, or kainic acid induces SCI via neuronal loss and subsequent inflammation [25]. Models of demyelination-induced SCI are also useful for evaluating the therapeutic effects of remyelination treatments. Drugs such as cuprizone and lysolecithin are frequently used to model multiple sclerosis [9], as these agents elicit partial demyelination when injected at specific sites in the spinal cord. In addition, animal models of demyelination-induced SCI can be developed using Theiler's murine encephalomyelitis virus and mouse hepatitis virus [26], allowing researchers to examine the efficacy of remyelination or Schwann cell grafting.

## 3. Therapeutic Approaches and Functional Recovery in Animal Models of Stem Cell Injury

**Figure 2:**
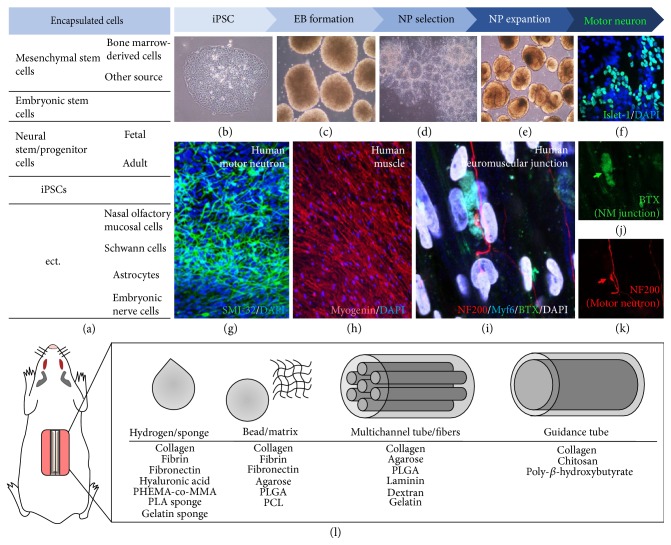
Multifaceted strategies for cell-based treatment. (a) List of cell types utilized in cell-based therapy for spinal cord injury. (b–k) Stem cell manipulation in various cell-based therapies. Induced pluripotent stem cells (iPSCs) can be generated from human somatic cells (b). Stem cells beginning to form embryoid body (c). Neural progenitors were isolated based on the neural rosette pattern and expanded in a culture dish (d, e). Following treatment with morphogenic agents, neural progenitors differentiate into many motor neurons (f, g). When motor neurons are cocultured with muscle fibers (h), the neuromuscular junction (i–k) can be detected on muscle fibers. (l) Scaffolds and biomaterials have developed for use in cell-based therapies. At a certain stage, stem cells can be transplanted alone or encapsulated in various scaffolds. PHEMA-co-MMA: poly(2-hydroxyethyl methacrylate-co-methyl methacrylate); PLA: polylactic acid; PLGA: poly(lactic-co-glycolic acid); PCL: polycaprolactone.

As there is currently no cure for deficits induced via SCI, various therapeutic strategies have been investigated using animal models. Although the efficacy of cell grafting has been examined in several trials over the past several decades [4], the use of multifaceted therapeutic approaches involving novel biomaterials or cell-encapsulated scaffolds has recently increased (Figure 2(c)). Interestingly, accumulating evidence suggests that the integration of several cell types and combined biomaterials promotes regeneration and functional improvement following SCI [27–30]. A summary of the relevant findings is provided in Table 1.

### 3.1. Mesenchymal Stem Cells (MSCs)/Bone Marrow Stromal Cells (BMSCs)

Since MSCs are easily obtained from autologous bone marrow or other sources, research regarding the use of MSC grafts or implantation of MSC-encapsulated scaffolds—which significantly reduce the immune response—has increased substantially in recent years. MSCs/BMSCs secrete significant levels of neurotrophic factors and can be transplanted via direct grafts, intravenously, or intrathecally. Recent evidence has suggested that these neurotrophic factors support CNS regeneration after injury. For example, direct infusion of neurotrophic factors such as brain-derived neurotrophic factor (BDNF) enhances neuronal survival and axonal growth in animal models of SCI [31, 32].

Fibrin, a representative fibrous protein that prevents blood clotting, is widely used to develop biopolymer scaffolds. Itosaka et al. [19] reported that, when BMSCs were supplied in conjunction with a fibrin matrix, model rats exhibited significant improvements in the recovery of neurological function, compared to when BMSCs were injected alone. In particular, remarkable improvements in motor function were observed (motor function test scale, BBB score = 15). The BBB scale is used to assess motor function, and BBB scores of 21 indicate complete recovery of motor function following injury. Additional studies have demonstrated that MSCs encapsulated with synthetic polymers (e.g., poly(lactic-co-glycolic acid) (PLGA) or N-(2-hydroxypropyl)-methacrylamide) attached to amino acid hydrogels (Arg-Gly-Asp (HPMA-RGD) hydrogels) can be implanted into the injured spinal cord cavity, resulting in increased tissue regeneration as well as significant improvements in functional recovery (BBB score = 10) [27, 33, 34]. During the first 1–6 months of recovery, structural scaffolds or hydrogels may promote tissue regeneration and functional improvement by assisting in the spread of healing factors within the damaged spinal cavity. Taken together, these results indicate that combined treatment involving cellular transplantation and biomaterial implants accelerates recovery from SCI, relative to the use of either treatment alone.

### 3.2. Neural Stem/Progenitor Cells (NSPCs)

Various studies have investigated the role of NSPCs—which can differentiate into neurons, astrocytes, or oligodendrocytes—in neuronal regeneration following SCI [35]. Research has indicated that transplanted NSPCs may promote functional recovery via neuroregenerative processes (e.g., remyelination) [28]. In 2002, Teng and colleagues [29] reported that neural stem cells (NSCs) implanted into the injured thoracic spinal cord using a PLGA scaffold (NSC-PLGA) produced increases in the number of corticospinal tract fibers passing through the injury epicenter and significantly improved motor function 10 weeks after implantation (BBB score = 10). Theses finding are in accordance with those of Du and colleagues [30], who also demonstrated the therapeutic effects of NSC-PLGA on tissue regeneration and motor function (BBB score = 4). Surprisingly, therapeutic effects increased by over twofold (BBB score = 8.8) when levels of neurotrophin-3 and tyrosine receptor kinase C expression were increased in NSCs. Researchers have consistently demonstrated the therapeutic effects of NSC-PLGA in both rat and primate models of SCI. Pritchard and colleagues [31] reported similar levels of recovery following NSC-PLGA implantation in African green monkeys (Babu score = 15). The Babu scale is used to evaluate function in primates, and the maximum score of 67 points is considered an indicative of complete recovery. However, the use of NSCs does not guarantee improvements in therapeutic outcomes. Although application of a chitosan or fibrin channel filled with NSPCs to the SCI cavity promotes the survival of NSCs, such animals exhibit no improvements in functional deficits [32, 36, 37]. These results indicate that therapeutic effects are only maximized when NSCs are administered in conjunction with certain biomaterials and structural scaffolds, such as the NSC-PLGA scaffold.

### 3.3. Induced Pluripotent Stem Cells (iPSCs)

Since iPSCs can be derived from somatic cells using reprogramming techniques, researchers have evaluated the utility of iPSCs in the treatment of various diseases. The use of iPSCs is advantageous in that these cells can be directed to develop into various cell types by modifying the differentiation protocol. NSCs can be induced via conversion from iPSCs (iPSC-NSCs) or via direct conversion of somatic cells (iNSCs). The embryoid body (EB) formation process can be used to generate NSCs from iPSCs (Figures 2(b) and 2(c)), following which therapeutic application of iPSC-NSCs is accomplished in a manner similar to that use for embryonic or adult NSCs, as described above (Figures 2(d) and 2(e)). Several reports have indicated that direct transplantation of iPSC-NSCs elicits therapeutic effects in both rat and primate models of SCI [35, 38, 39]. Nutt and colleagues [39] demonstrated that intraspinal grafting of iPSC-NSCs results in successful integration and neuronal differentiation within the injured cervical segment. Similarly, the application of iPSC-derived EBs (Figure 2(c); i.e., the prior status to NSCs) on a 3D fibrin-based scaffold (or a mixture composed of iPSC-NSCs and hydrogel) promotes neuronal survival and differentiation. These results indicate that iPSC-NSCs combined with biomaterials promote repair following SCI [40, 41]. Further studies have revealed that iNSCs also promote neuronal and functional recovery in a rat model of SCI, with therapeutic effects similar to those of normal NSCs [42, 43]. Notably, the application of iNSCs within a PLGA or PLGA-polyethylene glycol (PEG) scaffold also promotes tissue regeneration and functional recovery. However, the therapeutic effect of the combined PLGA-PEG scaffold is greater than that of the PLGA scaffold [44]. Various studies have also reported that transplantation of Schwann cells [45–47], astrocytes [48], nasal olfactory mucosal cells [49], dental pulp stem cells [50], and spinal cord cells [49] in conjunction with biomaterials significantly enhances repair and recovery following SCI [51]. Taken together, these results suggest that the most appropriate combination of cells and biomaterials should be chosen to maximize tissue regeneration and promote recovery of functional deficits following SCI.

Despite recent advancements, a number of safety issues have been associated with the transplantation of nonterminally differentiated cells, as both stem cells and iPSCs may increase the risk of developing iatrogenic teratomas or tumors. To address this issue, researchers have investigated the effects of therapeutic strategies involving the transplantation of several somatic cell types, such as fully differentiated astrocytes, Schwann cells, olfactory ensheathing cells, and spinal cord cells. As the area and severity of damage following SCI due to contusion or transection vary, cell-based therapy is more frequently applied than gene therapy or treatment with drugs/neurotrophic factors. For cellular therapy to be clinically effective, the grafted cells should enable the regeneration of axons and functional replacement of lost cells. Although recent clinical trials have extensively investigated such strategies [52], the inherent risks of cell-based therapy highlight the need to develop drug- or biomaterial-based strategies (e.g., methylprednisolone) for promoting axonal regeneration.

## 4. Behavioral Test Scales to Evaluate Motor Function

As previously mentioned, various animal models have been utilized in SCI research. Animal models can be classified by species, lesion methods, and injured segments of the spinal cord. Moreover, there are multifaceted therapeutic approaches to SCI repair, such as stem cell transplantation, administration of neurotrophic factors, or cell-encapsulated scaffold grafting. Given the enormous diversity of factors, it is difficult to compare and interpret the results of these studies. Thus, the need for universal, objective indices of functional impairment/improvement remains critical. Moreover, when evaluating the effects of various therapeutic strategies, both anatomical and functional recovery must be considered. Indeed, anatomical recovery defined based on increases in neuronal number, axonal regeneration, and reduction of lesion size remains inadequate without concomitant functional recovery. Based on this consideration, we propose that assessments of functional recovery be based on a unified numerical scoring system for each model (rodent/primate) and lesion site (cervical/thoracic). The BBB and Babu scales represent such unified indices for rats and primates, respectively. Summaries of testing categories and scoring indices are provided in Tables 2 and 3, respectively.

### 4.1. Rodent Thoracic Spinal Cord Injury Model

#### 4.1.1. BBB Scale

The BBB scale is a representative functional test administered following thoracic SCI injury [8, 11]. As mentioned previously, the development of thoracic SCI models is relatively easier and far less hazardous than the development of cervical models. In addition, rat models are more efficient than primate models due to the ease of operation and level of maintenance required in the animal facility. Thus, most SCI studies involve the use of rat models with injuries to the thoracic spinal cord. The BBB rating scale has long been utilized to assess thoracic SCI in rats. Thus, many studies report the BBB score only, which allows for sufficient estimation of the severity of functional impairments following injury. That is, the BBB scale functions as a unified index, allowing for straightforward evaluation and discussion of therapeutic effects. The BBB scale evaluates impairment based on locomotion in an open field, which allows researchers to observe voluntary movement, limb movement, trunk position, stepping patterns, and paw or tail position. Notably, scores can be evaluated at various stages of the recovery process: early, intermediate, and late phase after SCI. In the early phase, limb movements including hip, knee, ankle, and trunk positions are evaluated (BBB score = 0–8), followed by paw placement, stepping motion, and limb coordination in the intermediate phase (BBB score = 0–5). In the late phase, paw position—especially initial contact and lift-off behaviors—trunk instability, and tail position are used to evaluate the extent of functional recovery (BBB score = 0–8). A total maximum score of 21 points indicates normal function or complete recovery. However, as the BBB scale was developed to assess rat models of thoracic SCI (T_7_–T_9_) induced via contusion, there are several limitations regarding its general use in all rat models of SCI. In addition, rats may adapt to the open field environment, although this issue can be addressed by limiting testing time during rehabilitation.

#### 4.1.2. Louisville Swimming Scale (LSS)

The LSS was designed by Smith and colleagues [53] in 2006 for the assessment of hindlimb function during swimming. Although both the BBB and LSS can be used to assess impairments and recovery in rats with contusion-induced thoracic (T_9_) SCI, the LSS possesses some unique advantages over the BBB scale. As previously mentioned, adaptation and rehabilitation can vary depending on the housing conditions of the injured animals. Thus, swimming tests can be used to provide a novel environment and avoid the influence of retraining effects, allowing for more accurate assessment of locomotor capability. The LSS evaluates function based on the following five categories: hindlimb movement (LSS score = 0–4), alternation (score = 0–3), forelimb dependency (score = 0–4), trunk instability, and body angle (score = 0–6). A total maximum score of 17 points indicates normal function. Together with the BBB scale, the LSS has become widely utilized for evaluating impairments and recovery following thoracic injury in rats. However, as immobility behaviors can be influenced by emotional states/depression [54], swimming ability should be measured within a limited period.

#### 4.1.3. Combined Behavioral Score (CBS)

Developed in 1984 by Gale and colleagues [55], the CBS is the oldest rating scale for the assessment of deficits following contusion-induced thoracic SCI (T_8_). Function is assessed based on the following eight categories: hindlimb movement (CBS score = 0–45), toe spread and placement (score = 0–10), withdrawal reflexes following pain or pressure stimulation (score = 0–15), righting (score = 0–5), and maintenance of position on an inclined plane during increases in angle (beginning from 0°) (score = 0–15). In addition, somatosensory function is examined via the hot plate test, in which the latency to lick the forepaw and each hind paw when placed on a plate preheated to 50°C is scored (score = 0–5). Finally, in the swim test, the frequency of using the hindlimbs to swim and climb (score = 0–5) is recorded. A maximum CBS score of 100 points indicates severe paralysis in rats. However, as the range of CBS scores is quite wide, it is difficult to obtain consistent results from different observes. Due to these limitations, the ranges of scores in the more recently developed BBB and LSS are much narrower.

### 4.2. Rodent Cervical Spinal Cord Injury Model

#### 4.2.1. Martinez's Scale

Since cervical SCI is potentially life-threatening, scales for functional assessment following thoracic SCI are more developed than those for cervical SCI. Currently, only one such scale has been designed for use in models of cervical SCI: Martinez's scale [56]. In 2009, Martinez and colleagues developed this rating scale to examine functional recovery following cervical (C_4_) hemisection. The scale comprises eight categories that allow for separate evaluation of the forelimbs and hindlimbs: articular movement (score = 0–6), weight support pattern, digit position, and paw placement during stepping (score = 0–10 for all). Limb coordination and tail position are also evaluated (score = 0–4). A total maximum score of 20 points indicates full functional recovery. As cervical SCI affects both the forelimbs and hindlimbs, Martinez's scale is suitable for the precise assessment of functional recovery in all affected limbs. The primary advantage of Martinez's scale is that this rating system can be used to assess functional recovery following either thoracic or cervical SCI in rats. As previously mentioned, although various rating scales have developed, each possesses a distinct array of limitations. To address such limitations, other behavioral tests such as the catwalk test [57], gait analysis [58], grid walk test [59], and incline test [60] have been used in conjunction with rating scales to enable more precise evaluation of therapeutic effects. As cervical SCI can affect both forelimb and hindlimb function, testing procedures and categories should be divided according to the limb in animal models of cervical SCI. As scores on this scale can also be influenced by retraining effects, animal movement should be video-recorded during short sessions, followed by separate assessments of forelimb and hindlimb function.

### 4.3. Primate Thoracic Spinal Cord Injury Model

#### 4.3.1. Babu's Scale

The use of primate models in SCI research has recently increased. In 2002, Suresh Babu and colleagues [61] developed a rating scale designed to assess function in a primate model of thoracic SCI. Based on the CBS for rats, the modified scale was applied in Bonnet monkeys (*Macaca radiata*) following thoracic (T_12_–L_1_) hemisection. Babu's scale evaluates functional impairments based on the following two categories: reflex responses and locomotor behavior. Grasping, hopping, righting, and withdrawal reflexes due to extension, pressure, pain, and placement are evaluated (score = 0–22). In addition, gross locomotion on wide runways, narrow beams (score = 0–15), and grid runways (score = 0–20) is examined at various intervals. Finally, primates are subjected to a treadmill test at different speeds (score = 0–5) and levels of incline (score = 0–5). A total maximum score of 67 points on Babu's is considered an indicative of complete recovery. Although quite similar to the CBS for rats, Babu's scale is more detailed and includes additional criteria specific to primates.

### 4.4. Primate Cervical Spinal Cord Injury Model

#### 4.4.1. Nout's Scale

Nout and colleagues [62] designed a novel rating scale for the assessment of motor function in a rhesus monkey model of cervical (C_7_) SCI. Nout's scale is grossly divided into two categories: locomotion and hand function. Locomotion scores are based on general movement and trunk instability (score = 0–17), hindlimb movement, presence of weight support, presence of stepping, and extent of hindlimb (score = 0–21) and forelimb (score = 0–28) function. Due to the potential impairments associated with cervical SCI, hand function is also evaluated during object manipulation (score = 0–13), grasping, and digit movement (score = 0–8). A maximum score of 87 points on Nout's scale is considered an indicative of complete functional recovery.

Taken together, these findings indicate that accurate and unified criteria should be used to evaluate motor/sensory function in both rodent and primate models of SCI, in order to provide more objective assessments of therapeutic strategies. Such rating scales should be suitable for functional evaluation of the animal based on species and lesion type. Consistent use of such scales will lead more well-validated and effective SCI treatments.

In addition to rat/primate models, several studies have also evaluated therapeutic strategies in canine models of SCI [63, 64]. Behavioral assessments for canine models are based on the Tarlov scale [65], which was initially designed for use in rodents. This modified Tarlov scale evaluates motor coordination based on stepping behavior/regularity [64, 66].

## 5. Future Directions and Conclusions

In the present review, we summarized current evidence regarding animal models of SCI, cell-based therapeutic strategies, and rating scales used to evaluate motor function following SCI. Enhanced precision of SCI methods in recent years has reduced variations in the damage elicited during injury. The use of unified functional indices in conjunction with these more precise methods allows for sufficient estimation of the therapeutic effects of potential SCI treatments, without the need for additional descriptions. When the appropriate animal models and rating scales are chosen, our review suggests that the functional scores described in previous sections represent universal assessments of the animal's functional state. The use of defined animal models and suitable indices may also aid in identifying the most effective treatments and in enhancing the reproducibility of SCI research.

## Figures and Tables

**Table 1:** Animal models of stem cell injury (SCI) and multifaceted approaches to cellular therapies.

Reference	SCI animal model			Materials for scaffold	Applied cells	Recovery time	Tests for motor function	Functional outcome	BBB score
ltosaka et al., 2009	Rat	T_8_	Hemisection	Fibrin fibers	BMSC	4 weeks	BBB	Improved	15
Okuda, 2017		T_8_	Transection	Cell sheet		4 weeks	BBB	Improved	5
Kang et al., 2011		T_8-9_	Transection	PLGA scaffold	MSC	4 weeks	BBB	Improved	10
Yang, 2017		T_9-10_	Transection	PLGA scaffold		4 weeks	BBB	Improved	7
Hejcl et al., 2010		T_8-9_	Compression	HPMA-RGD hydrogel		35 weeks	BBB, plantar test	Improved	10
Hatami et al., 2009		T_10_	Hemisection	Type1 collagen droplet	NSC	5 weeks	BBB	Improved	19
Nomura et al., 2008		T_8_	Transection	Chitosan channels		12 weeks	BBB	No effect	9
Bozkurt et al., 2010		T_8_	Compression			9 weeks	BBB	No effect	11
Teng et al., 2002		T_9-10_	Hemisection	PLGA scaffold		10 weeks	BBB	Improved	10
Du et al., 2011		T_9-10_	Transection			8 weeks	BBB, Incline test	Improved	9
Johnson et al., 2010		T_9_	Hemisection	Fibrin scaffold		8 weeks	BBB, Grid walk	No effect	Data not shown
Ye, 2016		T_10_	Contrusion	Self-assembling peptide nanofiber		5 weeks	BBB	Improved	12
Mothes, 2013		T_2_	Compression	Hyaluronan-methy cellulose gel		9 weeks	BBB	No effect	12
Liu et al., 2015		T_10_	Transection	PLGA scaffold	iNSC	10 weeks	BBB	Improved	14
				PLGA-PEG scaffold		10 weeks	BBB	Improved	17
Olson et al., 2009		T_8-9_	Transection	PLGA scaffold	SC	4 weeks	BBB	No effect	1
Wang et al., 2011		T_10_	Transection	Gelform		8 weeks	BBB, incline test	Improved	8
Hurtado et al., 2006		T_9-10_	Transection	PDL tubular scaffold		6 weeks	BBB	No effect	7
Joosten et al., 2004		T_7-9_	Transection	Collagen gel	Astrocyte	4 weeks	BBB, grid, catwalk	No effect	13
Rochkind et al., 2006		T_7–8_	Transection	Dextran-gelatin tube	NOM, SCC	12 weeks	BBB	No effect	10
Zhang et al., 2016		T_9_	Contrusion	Chitosan scaffold	hDPSC	4 weeks	BBB	Improved	12
Pritchard et al., 2010	Primate	T_9_	Hemisection	PLGA scaffold	NSC	6 weeks	Babu scale	Improved	15
Nemati, 2013		C_5–_L_1_	Contrusion	Direct injection		49 weeks	Tarlov's scale	Improved	1.75

T: thoracic; C: cervical; L: lumbar; PLGA: poly(lactide-co-glycolide) acid; HPMA-RGD: N-(2-hydroxypropyl)-methacrylamide with attached amino acid sequences Arg-Gly-Asp; PDL: poly D-lactic acid; PLA: poly L-lactic acid; BMSC: bone marrow stromal cell; MSC: mesenchymal stem cells; NSC: neural stem cell; iNSC: induced neural stem cell; SC: Schwann cell; NOM: nasal olfactory mucosal cell; SCC: spinal cord cell; BBB: Basso, Beattie, and Bresnahan test scale.

**Table 2:** Rat models of spinal cord injury (SCI) and suitable test scales.

Target	Thoracic SCI								Cervical SCI				
Scale	BBB				LSS		CBS		Martinez				
Reference	Basso et al., 1995				Smith et al., 2006		Gale et al., 1985		Martinez et al., 2009				
Category	Recovery phase	Category I	Category II	Score	Swim ability	Score	Category	Score	Category	Forelimb	Score	Hindlimb	Score
	Early	Limb movement	Hip	0–2	HL movement	0–4	HL movement	0–45	Articular movement	Shoulder	0–2	Hip	0–2
			Knee	0–2	HL alternation	0–3	Toe spread	0–5		Elbow	0–2	Knee	0–2
			Ankle	0–2	FL dependency	0–4	Placing	0–5		Wrist	0–2	Ankle	0–2
		Trunk position	Side	0-1	Trunk instability	0–4	Withdrawal	0–15	Weight support	Stationary	0-1	Stationary	0-1
			Prop	0-1	Body angle	0–2	Righting	0–5		Active	0-1	Active	0-1
	Intermediate	Paw placement	Sweep	0–2			Inclined plane	0–15	Digit position		0–2		0–2
		Stepping	Dorsal	0-1			Hot plate	0–5	Paw placement at initial contact	Stepping	0–2	Stepping	0–2
			Plantar	0-1			Swim test	0–5					
		Limb coordination	Coordination	0-1					Paw orientation during lift off	Stepping	0–2	Stepping	0–2
	Late	Paw position	Initial contact	0–3									
			Lift off	0–3					Movement		0–2		0–2
		Trunk instability	Instability	0-1					Limb coordination		0–3		0–3
		Tail position	Up or down	0-1					Tail position		0-1		0-1
Maximum score (−normal)				21	(=normal)	17	(=severe)	100	Maximum score (=normal)		20		20

BBB: Basso, Beattie, and Bresnahan; LSS: Louisville swimming scale; CBS: combined behavioral score; HL: hindlimb; FL: Forelimb.

**Table 3:** Primate models of spinal cord injury (SCI) and suitable test scales.

Target	Thoracic SCI			Cervical SCI			
Scale	Babu			Nout			
Reference	Suresh Babu et al., 2000; bonnet monkey			Nout et al., 2012; rhesus monkeys			
Category 1	Category II	Category III	Score	Category I	Category II	Category III	Score
Reflex	Grasping		0–3	Locomotion	General	Forward movement	0–2
	Hopping	Low speed	0–3			Number of limbs used	0–4
		Medium speed	0–3			Number of perches reached	0–4
		High speed	0–3			Number of cups reached	0–5
	Righting		0–2			Truncal instability	0–2
	Extension withdrawal		0–2		Maximum score		17
	Pressure withdrawal		0–2		Hind limb	Extent of movements	0–8
	Pain withdrawal		0–2			Presence of weight support	0–3
	Placing		0–2			Presence of stepping	0–4
Runways	Runways	Wide runway	0–5			Ability and extent of use of the hind limb	0–6
		Narrow beam 1	0–5		Maximum score		21
		Narrow beam II	0–5		Forelimb	Extent of movements	0–8
	Grid runways	4 cm intervals	0–5			Presence of weight support	0–10
		5 cm intervals	0–5			Presence of stepping	0–4
		6 cm intervals	0–5			Ability and extent of use of the forelimb	0–6
		7 cm intervals	0–5		Maximum score		28
ect.	Treadmill test	Low speed	0–5	Hand function	Posture of the animal during object manipulation		0–5
		Medium speed			Use of the impaired hand for support and movement of the object		0–8
		High speed			Grasping method used		0–2
	Inclined plane test	Low degree	0–5		Extent of wrist and digit movements		0–6
		Medium degree			Maximum score		21
		High degree					
Maximum score (=normal)			67	Maximum score (=normal)			87

## References

[1] Ackery A., Tator C., Krassioukov A. (2004). A global perspective on spinal cord injury epidemiology. *Journal of Neurotrauma*.

[2] Singh A., Tetreault L., Kalsi-Ryan S., Nouri A., Fehlings M. G. (2014). Global prevalence and incidence of traumatic spinal cord injury. *Clinical Epidemiology*.

[3] Thuret S., Moon L. D., Gage F. H. (2006). Therapeutic interventions after spinal cord injury. *Nature Reviews Neuroscience*.

[4] Assinck P., Duncan G. J., Hilton B. J., Plemel J. R., Tetzlaff W. (2017). Cell transplantation therapy for spinal cord injury. *Nature Neuroscience*.

[5] Hodgetts S. I., Edel M., Harvey A. R. (2015). The state of play with iPSCs and spinal cord injury models. *Journal of Clinical Medicine*.

[6] McMahill B. G., Borjesson D. L., Sieber-Blum M., Nolta J. A., Sturges B. K. (2015). Stem cells in canine spinal cord injury--promise for regenerative therapy in a large animal model of human disease. *Stem Cell Reviews*.

[7] Cheriyan T., Ryan D. J., Weinreb J. H. (2014). Spinal cord injury models: a review. *Spinal Cord*.

[8] Basso D. M., Fisher L. C., Anderson A. J., Jakeman L. B., McTigue D. M., Popovich P. G. (2006). Basso mouse scale for locomotion detects differences in recovery after spinal cord injury in five common mouse strains. *Journal of Neurotrauma*.

[9] Denic A., Johnson A. J., Bieber A. J., Warrington A. E., Rodriguez M., Pirko I. (2011). The relevance of animal models in multiple sclerosis research. *Pathophysiology*.

[10] Koopmans G. C., Deumens R., Honig W. M., Hamers F. P., Steinbusch H. W., Joosten E. A. (2005). The assessment of locomotor function in spinal cord injured rats: the importance of objective analysis of coordination. *Journal of Neurotrauma*.

[11] Basso D. M., Beattie M. S., Bresnahan J. C. (1995). A sensitive and reliable locomotor rating scale for open field testing in rats. *Journal of Neurotrauma*.

[12] Gruner J. A. (1992). A monitored contusion model of spinal cord injury in the rat. *Journal of Neurotrauma*.

[13] Gensel J. C., Tovar C. A., Hamers F. P., Deibert R. J., Beattie M. S., Bresnahan J. C. (2006). Behavioral and histological characterization of unilateral cervical spinal cord contusion injury in rats. *Journal of Neurotrauma*.

[14] Liu L., Chi L. T., Tu Z. Q., Sheng B., Zhou Z. K., Pei F. X. (2004). Observation and establishment of an animal model of tractive spinal cord injury in rats. *Chinese Journal of Traumatology*.

[15] Zhang Y. P., Iannotti C., Shields L. B. (2004). Dural closure, cord approximation, and clot removal: enhancement of tissue sparing in a novel laceration spinal cord injury model. *Journal of Neurosurgery*.

[16] Heimburger R. F. (2005). Return of function after spinal cord transection. *Spinal Cord*.

[17] Freeman L. W. (1952). Return of function after complete transection of the spinal cord of the rat, cat and dog. *Annals of Surgery*.

[18] Hatami M., Mehrjardi N. Z., Kiani S. (2009). Human embryonic stem cell-derived neural precursor transplants in collagen scaffolds promote recovery in injured rat spinal cord. *Cytotherapy*.

[19] Itosaka H., Kuroda S., Shichinohe H. (2009). Fibrin matrix provides a suitable scaffold for bone marrow stromal cells transplanted into injured spinal cord: a novel material for CNS tissue engineering. *Neuropathology*.

[20] Li X., Yang Z., Zhang A., Wang T., Chen W. (2009). Repair of thoracic spinal cord injury by chitosan tube implantation in adult rats. *Biomaterials*.

[21] Watson B. D., Prado R., Dietrich W. D., Ginsberg M. D., Green B. A. (1986). Photochemically induced spinal cord injury in the rat. *Brain Research*.

[22] Gaviria M., Haton H., Sandillon F., Privat A. (2002). A mouse model of acute ischemic spinal cord injury. *Journal of Neurotrauma*.

[23] Hao J. X., Xu X. J., Aldskogius H., Seiger A., Wiesenfeld-Hallin Z. (1991). Allodynia-like effects in rat after ischaemic spinal cord injury photochemically induced by laser irradiation. *Pain*.

[24] Toumpoulis I. K., Anagnostopoulos C. E., Drossos G. E., Malamou-Mitsi V. D., Pappa L. S., Katritsis D. G. (2003). Does ischemic preconditioning reduce spinal cord injury because of descending thoracic aortic occlusion?. *Journal of Vascular Surgery*.

[25] Yezierski R. P., Liu S., Ruenes G. L., Kajander K. J., Brewer K. L. (1998). Excitotoxic spinal cord injury: behavioral and morphological characteristics of a central pain model. *Pain*.

[26] Das Sarma J. (2010). A mechanism of virus-induced demyelination. *Interdisciplinary Perspectives on Infectious Diseases*.

[27] Ropper A. E., Thakor D. K., Han I. (2017). Defining recovery neurobiology of injured spinal cord by synthetic matrix-assisted hMSC implantation. *Proceedings of the National Academy of Sciences of the United States of America*.

[28] Karimi-Abdolrezaee S., Eftekharpour E., Wang J., Morshead C. M., Fehlings M. G. (2006). Delayed transplantation of adult neural precursor cells promotes remyelination and functional neurological recovery after spinal cord injury. *The Journal of Neuroscience*.

[29] Teng Y. D., Lavik E. B., Qu X. (2002). Functional recovery following traumatic spinal cord injury mediated by a unique polymer scaffold seeded with neural stem cells. *Proceedings of the National Academy of Sciences of the United States of America*.

[30] Du B. L., Xiong Y., Zeng C. G. (2011). Transplantation of artificial neural construct partly improved spinal tissue repair and functional recovery in rats with spinal cord transection. *Brain Research*.

[31] Pritchard C. D., Slotkin J. R., Yu D. (2010). Establishing a model spinal cord injury in the African green monkey for the preclinical evaluation of biodegradable polymer scaffolds seeded with human neural stem cells. *Journal of Neuroscience Methods*.

[32] Bozkurt G., Mothe A. J., Zahir T., Kim H., Shoichet M. S., Tator C. H. (2010). Chitosan channels containing spinal cord-derived stem/progenitor cells for repair of subacute spinal cord injury in the rat. *Neurosurgery*.

[33] Hejcl A., Sedy J., Kapcalova M. (2010). HPMA-RGD hydrogels seeded with mesenchymal stem cells improve functional outcome in chronic spinal cord injury. *Stem Cells and Development*.

[34] Kang K. N., Lee J. Y., Kim D. Y. (2011). Regeneration of completely transected spinal cord using scaffold of poly(D,L-lactide-co-glycolide)/small intestinal submucosa seeded with rat bone marrow stem cells. *Tissue Engineering Part A*.

[35] Lu P., Woodruff G., Wang Y. (2014). Long-distance axonal growth from human induced pluripotent stem cells after spinal cord injury. *Neuron*.

[36] Nomura H., Zahir T., Kim H. (2008). Extramedullary chitosan channels promote survival of transplanted neural stem and progenitor cells and create a tissue bridge after complete spinal cord transection. *Tissue Engineering Part A*.

[37] Johnson P. J., Tatara A., McCreedy D. A., Shiu A., Sakiyama-Elbert S. E. (2010). Tissue-engineered fibrin scaffolds containing neural progenitors enhance functional recovery in a subacute model of SCI. *Soft Matter*.

[38] Kobayashi Y., Okada Y., Itakura G. (2012). Pre-evaluated safe human iPSC-derived neural stem cells promote functional recovery after spinal cord injury in common marmoset without tumorigenicity. *PLoS One*.

[39] Nutt S. E., Chang E. A., Suhr S. T. (2013). Caudalized human iPSC-derived neural progenitor cells produce neurons and glia but fail to restore function in an early chronic spinal cord injury model. *Experimental Neurology*.

[40] Montgomery A., Wong A., Gabers N., Willerth S. M. (2015). Engineering personalized neural tissue by combining induced pluripotent stem cells with fibrin scaffolds. *Biomaterials Science*.

[41] Saadai P., Wang A., Nout Y. S. (2013). Human induced pluripotent stem cell-derived neural crest stem cells integrate into the injured spinal cord in the fetal lamb model of myelomeningocele. *Journal of Pediatric Surgery*.

[42] Ma J., Li X., Yi B. (2014). Transplanted iNSCs migrate through SDF-1/CXCR4 signaling to promote neural recovery in a rat model of spinal cord injury. *Neuroreport*.

[43] Hong J. Y., Lee S. H., Lee S. C. (2014). Therapeutic potential of induced neural stem cells for spinal cord injury. *The Journal of Biological Chemistry*.

[44] Liu C., Huang Y., Pang M. (2015). Tissue-engineered regeneration of completely transected spinal cord using induced neural stem cells and gelatin-electrospun poly (lactide-co-glycolide)/polyethylene glycol scaffolds. *PLoS One*.

[45] Hurtado A., Moon L. D., Maquet V., Blits B., Jerome R., Oudega M. (2006). Poly (D,L-lactic acid) macroporous guidance scaffolds seeded with Schwann cells genetically modified to secrete a bi-functional neurotrophin implanted in the completely transected adult rat thoracic spinal cord. *Biomaterials*.

[46] Olson H. E., Rooney G. E., Gross L. (2009). Neural stem cell- and Schwann cell-loaded biodegradable polymer scaffolds support axonal regeneration in the transected spinal cord. *Tissue Engineering Part A*.

[47] Wang J. M., Zeng Y. S., Wu J. L., Li Y., Teng Y. D. (2011). Cograft of neural stem cells and Schwann cells overexpressing TrkC and neurotrophin-3 respectively after rat spinal cord transection. *Biomaterials*.

[48] Joosten E. A., Veldhuis W. B., Hamers F. P. (2004). Collagen containing neonatal astrocytes stimulates regrowth of injured fibers and promotes modest locomotor recovery after spinal cord injury. *Journal of Neuroscience Research*.

[49] Rochkind S., Shahar A., Fliss D. (2006). Development of a tissue-engineered composite implant for treating traumatic paraplegia in rats. *European Spine Journal*.

[50] Zhang J., Lu X., Feng G. (2016). Chitosan scaffolds induce human dental pulp stem cells to neural differentiation: potential roles for spinal cord injury therapy. *Cell and Tissue Research*.

[51] Shrestha B., Coykendall K., Li Y., Moon A., Priyadarshani P., Yao L. (2014). Repair of injured spinal cord using biomaterial scaffolds and stem cells. *Stem Cell Research & Therapy*.

[52] Gensel J. C., Donnelly D. J., Popovich P. G. (2011). Spinal cord injury therapies in humans: an overview of current clinical trials and their potential effects on intrinsic CNS macrophages. *Expert Opinion on Therapeutic Targets*.

[53] Smith R. R., Burke D. A., Baldini A. D. (2006). The Louisville swim scale: a novel assessment of hindlimb function following spinal cord injury in adult rats. *Journal of Neurotrauma*.

[54] Schechter M. D., Chance W. T. (1979). Non-specificity of “behavioral despair” as an animal model of depression. *European Journal of Pharmacology*.

[55] Gale K., Kerasidis H., Wrathall J. R. (1985). Spinal cord contusion in the rat: behavioral analysis of functional neurologic impairment. *Experimental Neurology*.

[56] Martinez M., Brezun J. M., Bonnier L., Xerri C. (2009). A new rating scale for open-field evaluation of behavioral recovery after cervical spinal cord injury in rats. *Journal of Neurotrauma*.

[57] Lankhorst A. J., ter Laak M. P., van Laar T. J. (2001). Effects of enriched housing on functional recovery after spinal cord contusive injury in the adult rat. *Journal of Neurotrauma*.

[58] Hamers F. P., Lankhorst A. J., van Laar T. J., Veldhuis W. B., Gispen W. H. (2001). Automated quantitative gait analysis during overground locomotion in the rat: its application to spinal cord contusion and transection injuries. *Journal of Neurotrauma*.

[59] Houweling D. A., Lankhorst A. J., Gispen W. H., Bar P. R., Joosten E. A. (1998). Collagen containing neurotrophin-3 (NT-3) attracts regrowing injured corticospinal axons in the adult rat spinal cord and promotes partial functional recovery. *Experimental Neurology*.

[60] Rivlin A. S., Tator C. H. (1978). Effect of duration of acute spinal cord compression in a new acute cord injury model in the rat. *Surgical Neurology*.

[61] Suresh Babu R., Muthusamy R., Namasivayam A. (2000). Behavioural assessment of functional recovery after spinal cord hemisection in the bonnet monkey (*Macaca radiata*). *Journal of the Neurological Sciences*.

[62] Nout Y. S., Ferguson A. R., Strand S. C. (2012). Methods for functional assessment after C7 spinal cord hemisection in the rhesus monkey. *Neurorehabilitation and Neural Repair*.

[63] Lee S. H., Chung Y. N., Kim Y. H. (2009). Effects of human neural stem cell transplantation in canine spinal cord hemisection. *Neurological Research*.

[64] Olby N. J., Risio L. D., Munana K. R. (2001). Development of a functional scoring system in dogs with acute spinal cord injuries. *American Journal of Veterinary Research*.

[65] Tarlov I. M., Klinger H., Vitale S. (1953). Spinal cord compression studies. I. Experimental techniques to produce acute and gradual compression. *A.M.A. Archives of Neurology and Psychiatry*.

[66] Olby N. J., Lim J. H., Babb K. (2014). Gait scoring in dogs with thoracolumbar spinal cord injuries when walking on a treadmill. *BMC Veterinary Research*.

